# Association of daily sitting time and coffee consumption with the risk of all-cause and cardiovascular disease mortality among US adults

**DOI:** 10.1186/s12889-024-18515-9

**Published:** 2024-04-17

**Authors:** Huimin Zhou, Jing Nie, Yanmei Cao, Linjing Diao, Xiaoli Zhang, Jiafu Li, Siyu Chen, Xu Zhang, Guochong Chen, Zengli Zhang, Bingyan Li

**Affiliations:** 1 Department of Nutrition and Food Hygiene, School of Public Health, Medical College of Soochow University, Suzhou, China; 2 State Key Laboratory of Radiation Medicine and Protection, School of Radiation Medicine and Protection, Soochow University, Suzhou, China; 3 Department of Occupational Medicine, The Affiliated Infectious Diseases Hospital of Soochow University, The Fifth People’s Hospital of Suzhou, Suzhou, China; 4 Department of Occupational and Environmental Health, School of Public Health, Medical College of Soochow University, Suzhou, China; 5 Department of Endocrinology, The Dushu Lake Hospital affiliated to Soochow University, 215000 Suzhou, Jiangsu China

**Keywords:** Sedentary behavior, Coffee Consumption, Mortality, National Health and Nutrition Examination Survey (NHANES)

## Abstract

**Background:**

Sedentary behavior has been demonstrated to be a modifiable factor for several chronic diseases, while coffee consumption is believed to be beneficial for health. However, the joint associations of daily sitting time and coffee consumption with mortality remains poorly understood. This study aimed to evaluate the independent and joint associations of daily sitting time and coffee intakes with mortality from all-cause and cardiovascular disease (CVD) among US adults.

**Methods:**

An analysis of a prospective cohort from the 2007–2018 National Health and Nutrition Examination Survey of US adults (*n* = 10,639). Data on mortality were compiled from interview and physical examination data until December 31, 2019. Daily sitting time was self-reported. Coffee beverages were from the 24-hour diet recall interview. The main outcomes of the study were all-cause and cardiovascular disease mortality. The adjusted hazard ratios [HRs] and 95% confidence intervals [CI] were imputed by Cox proportional hazards regression.

**Results:**

Among 10,639 participants in the study cohort, there were 945 deaths, 284 of whom died of CVD during the follow-up period of up to 13 years. Multivariable models showed that sitting more than 8 h/d was associated with higher risks of all-cause (HR, 1.46; 95% CI, 1.17–1.81) and CVD (HR, 1.79; 95% CI, 1.21–2.66) mortality, compared with those sitting for less than 4 h/d. People with the highest quartile of coffee consumption were observed for the reduced risks of both all-cause (HR, 0.67; 95% CI, 0.54–0.84) and CVD (HR, 0.46; 95% CI, 0.30–0.69) mortality compared with non-coffee consumers. Notably, joint analyses firstly showed that non-coffee drinkers who sat six hours or more per day were 1.58 (95% CI, 1.25–1.99) times more likely to die of all causes than coffee drinkers sitting for less than six hours per day, indicating that the association of sedentary with increased mortality was only observed among adults with no coffee consumption but not among those who had coffee intake.

**Conclusions:**

This study identified that sedentary behavior for more than 6 h/d accompanied with non-coffee consumption, were strongly associated with the increased risk of mortality from all-cause and CVD.

**Supplementary Information:**

The online version contains supplementary material available at 10.1186/s12889-024-18515-9.

## Introduction

Sedentary behavior is emerging as a modifiable risk factor for several chronic diseases [1–3]. Regardless of physical activity, prolonged sitting is independently associated with harmful health outcomes, including cancerous [4], metabolic [1, 5] and cardiovascular disease (CVD) [5–7]. Epidemiological evidences suggest that sedentary behavior is associated with the increased risk of all-cause mortality in the general population [1, 8, 9]. Prolonged sitting is also associated with an increased risk of CVD mortality, especially in people who do not achieve the recommended amount of physical activity [6, 7, 10, 11]. A study conducted on cancer survivors found that sedentary lifestyles and physical inactivity led to higher mortality for all-cause and cancer [12]. According to another study, sedentary behavior and physical inactivity were associated with higher all-cause and CVD mortality [13]. Prior research conducted in economically diverse settings, observed that, for the same high amounts of sitting time, a higher risk of all-cause and CVD mortality in low-income and lower-middle–income countries [7]. The study conducted in varying levels of frailty, found that, for people with low levels of frailty, sedentary time was not predictive of mortality, while people who were vulnerable (0.1 < frailty index score ≤ 0.2) or frail (frailty index score > 0.2), sedentary time was associated with higher mortality among those who were physically inactive [14]. Nevertheless, the impact of other life style combining with sedentary on mortality remain unknown.

Coffee, on the other hand, is one of the most widely consumed beverages in the world and among the population of the United States. Coffee intake varies dramatically in lifestyle and demographic factors, especially age [15, 16]. Moreover, coffee which is rich in bioactive substances such as caffeine, phenolic compounds, and minerals with a wide range of antioxidant, and anti-inflammatory effects, has been showed to improve insulin resistance and glucose metabolism [17, 18]. There is also growing evidence that drinking coffee significantly reduce morbidity and mortality from chronic diseases due to its powerful antioxidant properties [19, 20]. In cohort studies worldwide, coffee consumption has been associated with reduced mortality from all-cause and CVD [21–24]. According to the recent summaries on the coffee topic, we find that coffee is a complex mixture of compounds that may cause both harm and benefit [25–27]. As such, additional studies are needed to further elucidate the ideal way and dosage to consume coffee.

There’s plenty of evidence that sedentary behavior and coffee consumption are becoming more common [2, 3, 15, 16], and are independently associated with the risk of all-cause and CVD mortality. Coffee consumption is believed to have protective effect, while sedentary behavior seems to be disadvantageous. However, it remains unclear whether there is an interaction between sedentary behavior and coffee consumption, and the evidence on the impact of coffee intake on mortality in sedentary populations is limited.

In this study we aimed to go beyond the independent association has been conducted in in previous investigations since it is the first to evaluate the joint associations of daily sitting time and coffee intake with mortality from all-cause and CVD in the US nationally representative sample using data from the National Health and Nutrition Survey (NHANES).

## Methods

### Sampling method and implementation

The study used a nationally representative sample from NHANES of the US National Center for Health Statistics (NCHS). The survey has been conducted every two years since 1999 to measure Americans’ health and nutrition status. The NHANES protocols were all reviewed and approved by the National Center for Health Statistics Ethics Review Board, and all participants signed an informed consent form. In order to report this study, it followed the guidelines in the Strengthening the Reporting of Observational Studies in Epidemiology (STROBE) Statement (Additional file 1).

### Study population

**Fig. 1:**
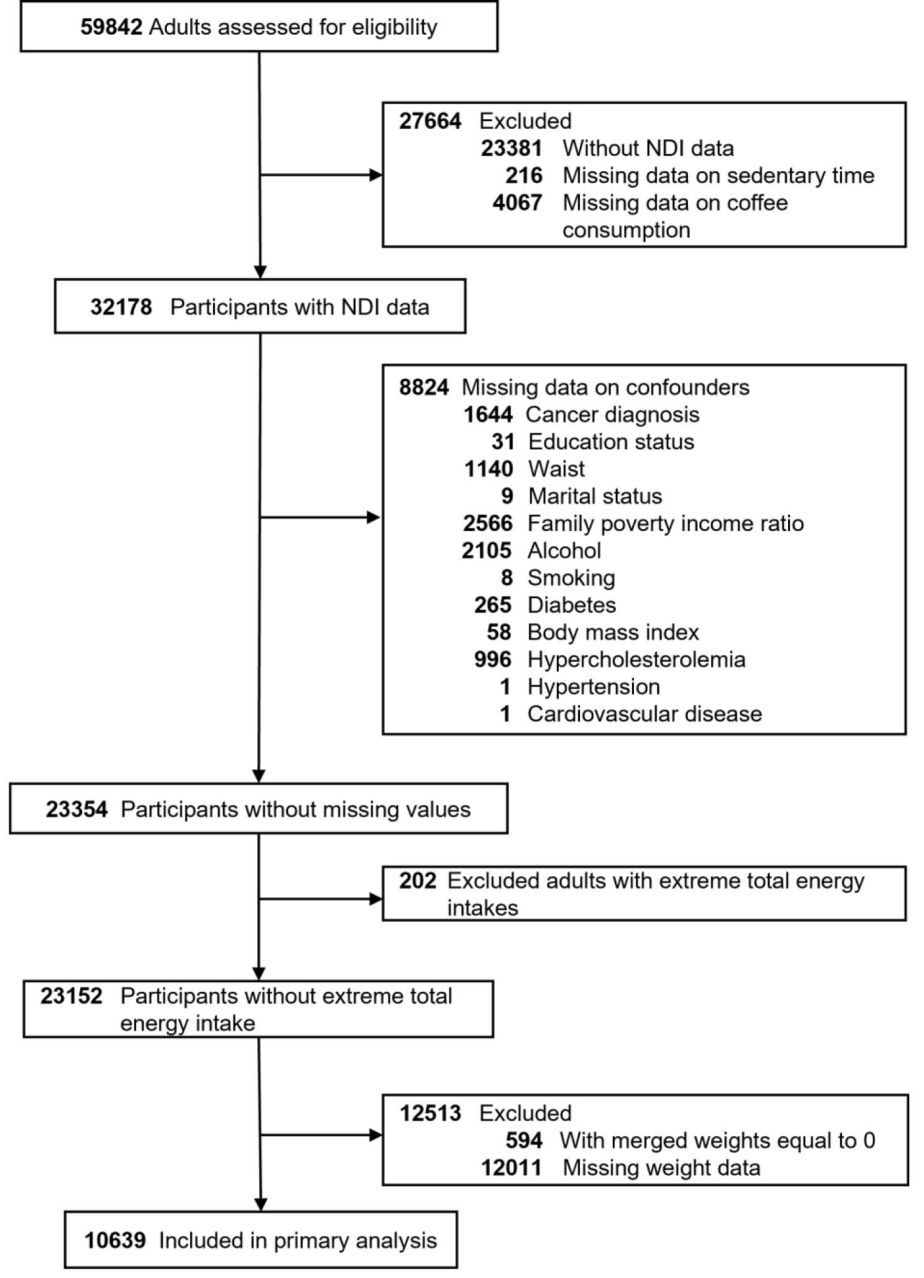
Flowchart of the screening process for the selection of eligible participants. Abbreviations: NDI, national death index. Extreme total energy intakes mean adults with total energy intakes of < 500 or 5000 kcal/day for women and < 500 or > 8000 kcal/day for men

In-person interviews and physical examinations and laboratory tests were conducted at a mobile testing center for each participant. Sociodemographic characteristics, lifestyle factors, and medical history of adults with available data on daily sitting time and coffee consumption for six NHANES cycles from 2007 to 2018 were examined and analyzed in this study. Excluding people that without mortality data that were linked to the National Death Index (NDI) [28]. and participants with unreliable total daily energy intake [29]. The initial baseline population was 59,842, with 27,664 missing data on exposure factors and outcome variables and 8824 missing data on covariates excluded, leaving 23,354 participants. Another 202 people with extreme total energy intake and 12,513 people with weight equal to 0 and missing weight data were excluded. Finally, 10,639 qualified participants with perfect information were included, as shown in Fig. 1.

### Daily sitting time and coffee consumption

Participant responses to the Global Physical Activity Questionnaire (GPAQ) were used to measure total sitting time each day, which has previously been validated to measure daily physical activity and sedentary behavior [30]. An in-person interview consisted of the following questions: “On a typical day, how much time do you usually spend sitting at school, at home, getting to and from places, or with friends, including time spent sitting at a desk, traveling in a car or bus, reading, playing cards, watching television, or using a computer?” Following recent studies [9, 12, 31], having converted the responses to hours per day (h/d), we classified them into four categories (0 to 4, 4 to 6, 6 to 8, and ≥ 8 h/d). We collected data on coffee consumption from the first 24-hour dietary recall interview, which provides the amount of each food and beverage consumed in grams. Food categorization scheme, What We Eat in America (WWEIA), categorized all foods consumed into 155 categories, including coffee consumption. [Using the food code number starting with the 921 in the United States Department of Agriculture (USDA) Food and Nutrient Database for Dietary Studies (FNDDS) to identify coffee beverages]. Based on total reported coffee intake, coffee consumers were identified as those participants who had consumed any amount of coffee on their recall days. The coffee consumers were divided into three groups by quartiles of coffee consumption (g/day), with non-consumers as an additional category. Thus, divided into four groups: (1) no coffee intake, (2) 0.23–326 g/day, (3) 326–540 g/day, and (4) ≥ 540 g/day.

### Ascertainment of mortality

As of December 31, 2019, NCHS provided mortality data linked to the NDI. A death’s underlying cause was recorded using the International Classification of Diseases and Related Health Problems, Tenth Revision (ICD-10). Mortality from cardiovascular disease was classified into heart disease (ICD-10 codes I00-I09, I11, I13, and I20-I51) or cerebrovascular disease (ICD-10 codes I60-I69) [32]. The follow-up duration is defined as the number of months between the interview date and the date of death, or until December 31, 2019 if no event occurred.

### Sociodemographic characteristics, lifestyle behaviors, and long-term conditions

As part of the self-report questionnaire, participants were asked to provide the following sociodemographic characteristics: sex, race and ethnicity, educational status, marital status, and family poverty income ratio (total family income divided by the poverty threshold; <1.3, 1.3 to < 3.5, ≥ 3.5) [12, 32]. Measurements of waist circumference, weight and height were taken during a physical examination, and the weight in kilograms divided by the height in meters squared was calculated as a body mass index (BMI) and categorized into three groups (< 25, 25.0-29.9, ≥ 30 kg/m^2^). Additionally, the waist circumference of men and women was divided into two groups according to whether they were abdominal obesity (men, ≥ 120 cm; women, ≥ 88 cm) [33]. There were a number of lifestyle factors assessed including smoking (never, former, moderate, heavy), alcohol consumption (never, former, mild, moderate, heavy), and the Healthy Eating Index-2015 (HEI-2015, derived from a 24-hour dietary recall interview) [34]. In the previous week, participants without physical activity (PA), with PA exceeding 0 min per week (min/week) but less than 150 min per week, and with PA exceeding 150 min per week were divided into categories inactive, insufficiently active, and sufficiently active, respectively [30, 35, 36].

Hypertension was either determined by NHANES-measured blood pressure (≥ 80 mm Hg [diastolic] or ≥ 130 mm Hg [systolic]) or self-reported by them if they received a diagnosis from a health professional, or determined by a self-reported history of prescription of antihypertensive drugs. In the NHANES, participants self-reported hypercholesterolemia when they received a diagnosis from a health professional or determined their total cholesterol level based on NHANES measurements (≥ 240 mg/dL; multiply by 0.0259 to convert to millimoles per liter). We collected data on cancer diagnoses during in-person interviews. Participants were asked, “Have you ever been told by a doctor or other health professional that you had a cancer or a malignancy of any kind?” Cancer survivor is someone who has answered yes to the question [37]. Self-reported history of CVD or diabetes of participants was collected who had received these diagnoses from a medical professional or were determined by a history of prescriptions for medications used to treat these diseases [32].

### Statistical analysis

Following the NHANES analysis guidelines, all analyses used sample weights with complex sampling designs to account for unequal selection probabilities, oversampling of certain subpopulations, and non-response adjustment. According to the NHANES tutorials, the new weights were calculated because we combined six cycles in the present study as shown in Supplementary Table 1 in Additional file 2. Weighted multivariable cox proportional hazards regression models were applied to estimate hazard ratios (HR) and 95% confidence intervals (CI) for the associations of daily sitting time and coffee consumption with mortality, respectively.

To examine the joint associations, prolonged sitting is defined as sitting for more than 6 h per day [6, 25] and participants were grouped according to sitting time and coffee consumption to estimate the risk of mortality using weighted multivariate Cox proportional hazards regression model with adjustment for age, sex, race and ethnicity, educational attainment, household poverty-to-income ratio, BMI, waist circumference, marital status, smoking status, alcohol consumption, HEI-2015 score, hypertension, hypercholesterolemia, history of diabetes, CVD, cancer diagnosis and PA. People who spent less than 6 h of sedentary time and consumed coffee were used as the reference group. Multiple cox regression analyses were also used to verify an interaction scale for daily sitting time × coffee consumption. We also performed subgroup analyses by potential confounders. To perform sensitivity analyses, deaths that occurred during the first two years of follow-up were excluded [38].

The descriptive statistics are expressed as weighted means ± standard errors and frequency (weighted percentages) for continuous and categorical variables. All analyses were conducted with the “survey” package in R 4.3.1. Statistical tests were 2-sided, and statistical significance was set at *P* < 0.05.

## Results

### Baseline characteristics

Of 10,639 participants (weighted population, 178,944,896; weighted mean [SE] age, 47.1 [0.3] years; 50.0% female) in the study cohort, and 4786 (45.0%) Non-Hispanic White, 2023 (19.0%) Non-Hispanic Black, 2693 (25.3%) Hispanic, and 1137 (10.7%) were individuals of “other” race or ethnicity, including American Indian/Native Alaskan/Pacific Islander, Asian, and multiracial. People who sit more than 6 h/d were more likely to be non-Hispanic white, had an education level above high school. Meanwhile, they had higher waist circumference, and were more likely to have abdominal obesity, as well as BMI is more likely to be 30 and above (Table 1). In addition, coffee drinkers were more likely to be older, non-Hispanic white, and educated above high school (Additional file 2: Supplementary Table 2). Only 52.1% of US adults were coffee consumers. Almost half adults (48.1%) reported sitting for more than 6 h/d. Importantly, 23.0% of US adults reported both sitting for more than 6 h/d and no coffee consumption (Additional file 2: Supplementary Table 3).

**Table 1:** Sample Size ^a^ and baseline characteristics of the study population by daily sitting time

Variable	Daily sitting time				
	Total	<4 h	4 to 6 h	6 to 8 h	>8 h
Age, y	47.1 (0.3)	45.4 (0.4)	47.9 (0.5)	47.7 (0. 6)	47.4 (0.4)
Sex					
Female	5316 (50.0)	1468 (49.1)	1288 (51.6)	827 (50.5)	1733 (49.3)
Male	5323 (50.0)	1477 (50.9)	1290 (48.4)	848 (49.5)	1708 (50.7)
Race and ethnicity					
Hispanic	2693 (25.3)	1099 (21.8)	676 (14.2)	336 (10.4)	582 (8.5)
Non-Hispanic White	4786 (45.0)	1069 (60.7)	1134 (67.7)	836 (72.8)	1747 (74.6)
Non-Hispanic Black	2023 (19.0)	517 (10.9)	518 (11.0)	302 (9.4)	686 (10.0)
Other ^b^	1137 (10.7)	260 (6.6)	250 (7.1)	201 (7.4)	426 (6.9)
Educational attainment					
< High school	2378 (22.4)	959 (22.3)	616 (15.9)	312 (13.3)	491 (9.6)
High school	2410 (22.7)	733 (28.1)	621 (24.7)	392 (22.7)	664 (17.6)
> High school	5851 (55.0)	1253 (49.6)	1341 (59.4)	971 (64.0)	2286 (72.8)
Marital status					
Married	5571 (52.4)	1543 (55.4)	1364 (57,0)	869 (54.3)	1795 (56.4)
Never married	1908 (17.9)	476 (16.8)	450 (17.6)	325 (20.9)	657 (18.5)
Divorced	1175 (11.0)	321 (10.2)	273 (9.5)	166 (9.1)	415 (11.4)
other	1985 (18.7)	605 (17.6)	491 (15.9)	315 (15.7)	574 (13.7)
HEI-2015 ^c^	50.43 (0.3)	50.60 (0.4)	49.69 (0.4)	50.12 (0.5)	50.95 (0.4)
Family poverty income ratio					
<1.3	3223 (30.3)	1111 (28.0)	810 (21.9)	495 (20.1)	807 (15.3)
1.3–3.5	4061 (38.2)	1204 (40.5)	1037 (39.1)	620 (36.2)	1200 (31.2)
≥3.5	3355 (31.5)	630 (31.5)	731 (39.1)	560 (43.8)	1434 (53.5)
BMI, kg/m^2^					
<25	3037 (28.6)	890 (33.1)	753 (30.7)	482 (29.1)	912 (27.1)
25-29.9	3554 (33.4)	1059 (34.7)	893 (33.8)	519 (31.3)	1083 (32.4)
≥30	4048 (38.1)	996 (32.2)	932 (35.4)	674 (39.6)	1446 (40.5)
Waist circumference	99.44 (0.3)	96.88 (0.4)	98.68 (0.5)	100.28 (0.6)	101.25 (0.5)
Abdominal obesity ^d^					
Yes	6077 (57.1)	1554 (50.0)	1466 (57.2)	979 (58.3)	2078 (60.0)
Alcohol use					
Never	1369 (12.9)	453 (12.0)	312 (10.0)	220 (10.3)	384 (8.8)
Former	1623 (15.3)	435 (12.0)	423 (13.7)	269 (12.9)	496 (11.3)
Mild	3789 (35.6)	913 (33.0)	918 (38.5)	622 (39.4)	1336 (41.6)
Moderate	1664 (15.6)	432 (16.0)	387 (16.8)	241 (16.3)	604 (20.4)
Heavy	2194 (20.6)	712 (27.0)	538 (21.0)	323 (21.1)	621 (17.9)
Smoking status					
Never	5886 (55.3)	1690 (54.7)	1394 (55.1)	904 (54.5)	1898 (57.2)
Former	2646 (24.9)	668 (24.7)	638 (23.0)	434 (26.6)	906 (27.0)
Now	2107 (19.8)	587 (20.6)	546 (21.8)	337 (18.9)	637 (15.8)
Diabetes					
Yes	1474 (13.9)	389 (8.4)	346 (9.8)	226 (9. 9)	513 (11.4)
Hypertension					
Yes	5585 (52.5)	1448 (44.0)	1381 (49.8)	906 (48.6)	1850 (48.8)
Cardiovascular disease					
Yes	1138 (10.7)	255 (7.0)	254 (8.2)	208 (10.0)	421 (9.3)
Hypercholesterolemia					
Yes	1304 (12.3)	376 (12.4)	353 (14.7)	202 (11.9)	373 (11.4)
Cancer diagnosis					
Yes	980 (9.2)	203 (7.5)	239 (9.9)	186 (10.2)	352 (10.0)
PA, min/wk					
None (inactive)	2567 (24.1)	571 (14.9)	526 (16.6)	420 (20.5)	1050 (26.0)
0 to < 150 (insufficiently active)	1463 (13.8)	333 (10.0)	308 (11.7)	227 (12.0)	595 (17.1)
≥ 150 (active)	6609 (62.1)	2041 (75.2)	1744 (71.8)	1028 (67.5)	1796 (56.9)
Coffee consumption					
None	5095 (47.9)	1420 (47.3)	1225 (46.6)	800 (48.2)	1650 (44.4)
Q1 ^f^	1850 (17.4)	579 (16.4)	434 (13.9)	298 (14.2)	539 (14.1)
Q2 ^f^	1863 (17.5)	495 (17.1)	477 (18.9)	281 (16.4)	610 (19.0)
Q3 ^f^	1831 (17.2)	451 (19.2)	442 (20.6)	296 (21.2)	642 (22.4)

Abbreviations: BMI, body mass index (calculated as weight in kilograms divided by height in meters squared); PA, physical activity; h, hours; min/wk, minutes per week; NHANES, the National Health and Nutrition Examination Survey

The descriptive statistics are expressed as mean ± standard deviation and number (percentage) for continuous and categorical variables

^**a**^ Weighted to be nationally representative

^**b**^ Including American Indian/Alaska Native/Pacific Islander, Asian, and multiracial

^**C**^ HEI-2015 (Healthy Eating Index-2015, which is derived from a 24-hour dietary recall interview, and measures the quality of a person’s overall diet from 0 to 100 [worst to best])

^**d**^ Abdominal obesity, men and women were divided into two groups based on their waist circumferences (men, ≥ 120 cm; women, ≥ 88 cm)

^**f**^ Coffee consumption(g/day) of coffee drinkers was divided into three groups (Q1, Q2 and Q3) by quartile. Q1, Q2 and Q3 are < 326, 326–540, > 540, respectively

### Multivariate cox proportional models for independent association of sedentary and coffee consumption on mortality

As shown in Table 2, during the follow-up period of up to 13 years (median, 6.5 years), there were 945 deaths, 284 of them died of CVD. Adults who sit for long periods (> 8 h/d) have an increased risk of all-cause and CVD mortality. After adjusting for covariates and PA, HRs for all-cause and CVD mortality among individuals sitting more than 8 h/d, compared with those sitting less than 4 h/d, were 1.46 (95% CI, 1.17–1.81) and 1.79 (95% CI, 1.21–2.66), respectively. Meanwhile, compared with non-coffee drinkers, all-cause and CVD mortality risks in the highest quartile of coffee intake were 0.67 (95% CI, 0.54–0.84) and 0.46 (95% CI, 0.30–0.69), respectively (Table 2). Furthermore, among coffee consumers, the HRs for CVD mortality significantly decreased as coffee consumption increased.

**Table 2:** Association of daily sitting time and coffee consumption with all-cause and cardiovascular disease mortality

Mortality outcome	Death/No.	Weighted death (%)	Hazard ratio (95% CI)		
			Model 1	Model 2	Model 3
**All causes**					
Daily sitting time					
< 4 h	199/2945	2,059,972 (4.8)	1 [Reference]	1 [Reference]	1 [Reference]
4 to 6 h	218/2578	2,599,351 (6.1)	1.16 (0.92, 1.47)	1.13 (0.89, 1.44)	1.11 (0.88, 1.41)
6 to 8 h	164/1675	1,860,378 (6.4)	1.15 (0.88, 1.50)	1.09 (0.83, 1.43)	1.06 (0.80, 1.39)
≥ 8 h	364/3441	4,616,467 (7.1)	1.61 (1.33, 1.94)	1.58 (1.28, 1.95)	1.46 (1.17, 1.81)
***P*** **for trend**	NA	NA	< 0.001	< 0.001	< 0.001
Coffee consumption(g/d)					
Q0^a^	344/5095	4,033,369 (4.9)	1 [Reference]	1 [Reference]	1 [Reference]
Q1^b^	219/1850	2,293,754 (8.8)	0.93 (0.76, 1.14)	0.90 (0.73, 1.12)	0.89 (0.71, 1.11)
Q2^b^	180/1863	2,203,843 (6.8)	0.79 (0.64, 0.99)	0.79 (0.64, 0.99)	0.81 (0.65, 1.01)
Q3^b^	202/1831	2,605,203 (6.9)	0.79 (0.63, 0.98)	0.68 (0.55, 0.84)	0.67 (0.54, 0.84)
***P*** **for trend**	NA	NA	0.02	< 0.001	< 0.001
**CVD**					
Daily sitting time					
< 4 h	52/2945	461,929 (1.1)	1 [Reference]	1 [Reference]	1 [Reference]
4 to 6 h	70/2578	848,548 (2.0)	1.59 (1.01, 2.51)	1.48 (0.93, 2.37)	1.45 (0.90, 2.33)
6 to 8 h	47/1675	480,049 (1.7)	1.27 (0.76, 2.13)	1.17 (0.69, 1.97)	1.12 (0.67, 1.86)
≥ 8 h	115/3441	1,411,401 (2.2)	2.20 (1.52, 3.17)	2.01 (1.34, 3.01)	1.79 (1.21, 2.66)
***P*** **for trend**	NA	NA	< 0.001	0.001	0.01
Coffee consumption(g/d)					
Q0^a^	115/5095	1,378,022 (1.7)	1 [Reference]	1 [Reference]	1 [Reference]
Q1^b^	68/1850	630,541 (2.4)	0.72 (0.51, 1.02)	0.71 (0.50, 1.00)	0.70 (0.49, 0.99)
Q2^b^	53/1863	613,430 (1.9)	0.61 (0.40, 0.93)	0.62 (0.40, 0.96)	0.64 (0.42, 0.99)
Q3^b^	48/1831	579,934 (1.5)	0.50 (0.32, 0.77)	0.45 (0.29, 0.68)	0.46 (0.30, 0.69)
***P*** **for trend**	NA	NA	< 0.001	< 0.001	< 0.001

Abbreviations: h, hours; g/d, grams per day; NA, not applicable; NHANES, the National Health and Nutrition Examination Survey

**Model 1**: adjusted for age

**Model 2**: multivariable model additionally adjusted for sex (male/female), race and ethnicity (non-Hispanic Black, Hispanic, non-Hispanic White, other race or ethnicity [including American Indian/Alaska Native/Pacific Islander, Asian, multiracial]), education level (< high school, high school, > high school), BMI (< 25, 25-29.9, > 30), waist circumference, marital status (married, divorced, unmarried), smoking status (never, former, current), alcohol use (never, ever, mild, moderate, heavy) and Healthy Eating Index-2015 score, family poverty income ratio (< 1.30, 1.30–3.49 or > 3.5), hypertension (yes/no), history of diabetes (yes/no), hypercholesterolemia (yes/no), cardiovascular disease (yes/no) and history of cancer diagnosis (yes/no)

**Model 3**: additionally adjusted for physical activity (PA)

^**a**^ Q0 means non-coffee consumers

^**b**^ Coffee consumption(g/day) of coffee drinkers was divided into three groups (Q1, Q2 and Q3) by quartile. Q1, Q2 and Q3 are < 326, 326–540, > 540, respectively

### Multivariate cox proportional models for joint association of sedentary and coffee consumption with mortality

A specific finding of the study was that non-coffee drinkers who sat six hours or more per day were 1.58 (95% CI, 1.25–1.99) times more likely to die of all causes mortality than coffee drinkers sitting for less than six hours per day. Similar association was observed for deaths caused by CVD. Notably, the association between sedentary and increased mortality was only observed in adults who did not drink coffee, but not in adults who drank coffee (Table 3). In the stratified analysis by coffee intake, longer sitting time was associated with elevated risks of all-cause mortality among those who were non-coffee consumers, while sitting time was not associated with all-cause mortality among adults with the highest quartile of coffee intake (Additional file 2: Supplementary 4). In sensitivity analyses, the results remained similar after excluding deaths during the first two years (Additional file 2: Supplementary Table 5; Supplementary Table 6).

**Table 3:** Joint association of daily sitting time and coffee consumption with all-cause and cardiovascular disease mortality

Mortality outcome	Sedentary time	Death/No.	Weighted death (%)	Hazard ratio (95% CI)		
				Model 1	Model 2	Model 3
**All causes**						
Coffee consumers	Sitting time, < 6 h/d	272/2878	2,904,562 (6.4)	1 [Reference]	1 [Reference]	1 [Reference]
	Sitting time, ≥ 6 h/d	329/2666	4,198,239 (8.2)	1.40 (1.12, 1.74)	1.31 (1.05, 1.63)	1.22 (0.97, 1.54)
Non-consumers	Sitting time, < 6 h/d	145/2645	1,754,762 (4.4)	1.31 (1.02, 1.69)	1.30 (1.01, 1.67)	1.28 (0.99, 1.65)
	Sitting time, ≥ 6 h/d	199/2450	2,278,607(5.3)	1.58 (1.24, 2.01)	1.66 (1.31, 2.09)	1.58 (1.25, 1.99)
**CVD**						
Coffee consumers	Sitting time, < 6 h/d	73/2878	713,171 (1.6)	1 [Reference]	1 [Reference]	1 [Reference]
	Sitting time, ≥ 6 h/d	96/2666	1,110,734 (2.2)	1.54 (1.15, 2.06)	1.39 (1.01, 1.93)	1.27 (0.91, 1.75)
Non-consumers	Sitting time, < 6 h/d	49/2645	597,306 (1.5)	1.90 (1.17, 3.09)	1.85 (1.13, 3.04)	1.78 (1.08, 2.95)
	Sitting time, ≥ 6 h/d	66/2450	780,716 (1.8)	2.34 (1.53, 3.58)	2.29 (1.46, 3.59)	2.10 (1.34, 3.29)

Abbreviations: h/d, hours per day; NHANES, the National Health and Nutrition Examination Survey

**Model 1**: adjusted age

**Model 2**: multivariable model additionally adjusted for sex (male/female), race and ethnicity (non-Hispanic Black, Hispanic, non-Hispanic White, other race or ethnicity [including American Indian/Alaska Native/Pacific Islander, Asian, multiracial]), education level (< high school, high school, > high school), BMI (< 25, 25-29.9, > 30), waist circumference, marital status (married, divorced, unmarried), smoking status (never, former, current), alcohol use (never, ever, mild, moderate, heavy) and Healthy Eating Index-2015 score, family poverty income ratio (< 1.30, 1.30–3.49 or > 3.5), hypertension (yes/no), history of diabetes (yes/no), hypercholesterolemia (yes/no), cardiovascular disease (yes/no) and history of cancer diagnosis (yes/no)

**Model 3**: additionally adjusted for PA

### Subgroup analysis

In the subgroup analyses based on daily sitting time, the association between daily sitting time and the risk of all-cause mortality was similar for most strata (*P* = 0.05–0.996) (Additional file 2: Supplementary Table 8). Significant interactions were observed only for age (*P* = 0.02). The risk of all-cause mortality was significantly higher in people over 65 years of age who were sedentary for more than 8 h per day (HR 1.69, 95% CI 1.25–2.28,*P* < 0.0001). When the cox regression model was constructed, the daily sitting time was less than 4 h as the reference group, and the remaining three groups were set as three dummy variables to enter the regression model for trend testing. The results showed that among people over 65 years of age, women, people with abdominal obesity, former drinkers, former smokers, no physical activity and insufficiently physical activity, the HR value of all-cause mortality risk increased with the increase of daily sitting time. The trend test P-trend < 0.05 indicates that the upward trend is statistically significant.

In the subgroup analyses based on coffee consumption, the association between coffee consumption and the risk of all-cause mortality was similar for most strata (*P* = 0.18–0.996) (Additional file 2: Supplementary Table 9). Significant interactions were observed only for race and ethnicity (*P* = 0.02). The risk of all-cause mortality was significantly higher in Non-Hispanic Black of coffee consumers (HR 0.57, 95%CI 0.40–0.81, *P* = 0.002).

## Discussion

In the US nationally representative population from the NHANES, 52.1% of adults were coffee consumers, and 48% reported sitting for more than 6 h/d. Altogether, 23% of adults reported no coffee consumption with sitting more than 6 h/d. During as many as 13 years of follow-up, we found a significant association between adults with sitting more 8 h/d with the increased risk of all-cause and CVD mortality. Meanwhile, all-cause mortality was significantly decreased in the population with highest quintile of coffee consumption, while CVD mortality was reduced for any amount of coffee intake. Notably, the results of the joint analysis identified that sedentary behavior for more than 6 h/d accompanied with non-coffee consumption, were strongly associated with the increased risk of mortality from all-cause and CVD.

The link between sedentary behavior and poor survival outcomes has been demonstrated in some studies [9, 39, 40], as well as this study. The inflammation is considered to be one of the important mechanisms. One research has indicated that prolonged and uninterrupted sitting appears to impair glucose metabolism and increase inflammation [41]. Sedentary behavior is a crucial and independent predictor of inflammation, as it induces pro-inflammatory markers while reducing anti-inflammatory markers [42]. Researchers have found that sedentary time is positively correlated with C-reactive protein (CRP) levels, independent of physical activity and obesity [43]. In some prospective cohort studies, elevated CRP was positive associated with the adjusted mortality for all-cause [44–46] and CVD [45]. Additionally, previous studies had shown that sedentary behavior alters the metabolism of skeletal muscle [47] and for each hour more spent sitting or lying in a prone position during waking hours, metabolic risks increased by 39% [48, 49]. A review suggested that physical inactivity affected an individual’s phenotype, organ systems, and diseases, including behavior, central nervous system, cardiorespiratory fitness (CRF), skeletal muscle, metabolism, bone, immunity, adipose tissue, digestion [50]. Regarding the impact of CRF on mortality, CVD mortality increased 19% and all-cause mortality increased 15% for every 1-Metabolic Equivalent(MET)loss in 14,345 men aged 44 after an average of 11.4 years from the Aerobics Center Longitudinal Study [51]. This association has persisted in other studies. The result from a classic double-level bus study in 1953, showed that the conductor had a 30% reduction in the incidence of coronary heart disease compared to drivers when drivers sat and conductors walked up and down the stairs. Furthermore, conductors were older when they developed the disease, which was less severe with lower fatality rates than the drivers [52]. Physical inactivity itself is a direct cause of accelerating cardiovascular, shortening healthy life span, and lowering the age of onset of the first chronic disease.

The benefits of coffee consumption in improving overall survival in adults compared to sedentary behavior are manifold. Coffee consumption reduces the risk of metabolic syndrome, which aggravates inflammation [53, 54]. An inverse relationship between coffee consumption with all-cause and CVD mortality has been found in the adult in many studies [24, 55–59]. Our results, consistent with the above study, observed that the highest quartile of coffee consumption was inversely correlated with all-cause mortality compared with non-coffee drinkers (HR, 0.67; 95% CI, 0.54–0.84). However, the mechanism by which coffee reduces mortality from all causes is unclear. Over 1,000 compounds can be found in coffee. The most popular are caffeine, chlorogenic acid, trigonoid, melanoids, caffeic acid, cafestol, and kahweol, and polyphenols, which have anti-inflammatory properties [60, 61]. Unfiltered coffee containing cafestol and kahweol, prevent inflammation-related diseases by reducing inflammatory responses [62]. There are many important biochemical pathways involved in inflammation that are regulated by chlorogenic acid, another very important compound in coffee [63–65]. It is believed that coffee consumption increases the level of sirtuin-1 (SIRT1), a protein with anti-inflammatory properties [66]. According to cross-sectional and prospective cohort studies, intake of certain polyphenols (flavonoid classes, in particular) reduces CVD risk [67, 68].

By performing an interaction test, we find that the interaction between sedentary behavior and coffee consumption was negative, but not significant, indicating that there was no significant negative multiplication model interaction between the two factors on the risk of all-cause mortality. Interaction is a statistical term that describes the combined effect of two or more exposures in the data, and they cannot be directly said to have a biological sense of interaction. Rothman pointed out that the product terms in logistic or cox regression models were not statistically significant and did not indicate biological interaction between the two factors [69]. Subsequently, more prospective studies can be conducted to verify whether there is an interaction between sedentary and coffee consumption by expanding the sample size or by targeting specific types of coffee consumption. At the same time, in joint analyses of this study, prolonged sitting was associated with increased mortality only among adults who did not consume coffee, but not among those who consumed coffee. The effect of sedentary behavior on mortality risk varies among different populations and more research is also needed to investigate.

In the present study, when participants were stratified according to potential confounders, no significant interactions were observed for coffee consumption, sex, abdominal obesity, BMI, educational attainment, race, marital status, alcohol, smoke, family poverty income ratio, diabetes, hypertension, hypercholesterolemia, CVD, cancer diagnosis and PA. But interactive effects of age by daily sitting time (*P* = 0.02), and race and ethnicity by coffee consumption (*P* = 0.02) on all- cause mortality risks, were observed respectively. However, little is known about the mechanism underlying the interaction between daily sitting time and age, as well as between coffee consumption and race. Sedentary behavior increases the risk of adverse health outcomes in older adults, similar to the results of a study in older women [70]. Therefore, we should consider age when evaluating the relationship between daily sitting time and all-cause mortality, and we also should consider race and ethnicity when evaluating the relationship between the coffee consumption and all-cause mortality.

Overall, sedentary behavior has been showed to increase the risk of all-cause and CVD mortality, conversely, coffee intake has been observed to reduce the risk. To our knowledge, this is the first study to investigate the joint associations of daily sitting time and coffee consumption with all-cause and CVD mortality. Interestingly, both sedentary behavior and coffee consumption have been demonstrated to display an effect on inflammation-related biomarkers. Therefore, well-designed prospective cohort studies should be conducted to determine the impact of coffee intake on health benefits in sedentary populations.

### Strengths and limitations

Daily sitting time and coffee intakes were derived from the database NHANES, which is a large prospective cohort with well-designed and validated protocols. Furthermore, a variety of confounding factors were considered, including BMI and abdominal obesity. However, there are also several limitations need to be addressed. Firstly, daily sitting time were self-reported and could not accurately present actual sitting time. In the stage of questionnaire collection and sorting, subject information bias cannot be ignored, and these biases may affect the results of statistical analysis, suggesting that a prospective multicenter study is needed. Secondly, we examined the association of quantities of coffee consumption with outcome, but did not conduct the impact of specific coffee types on mortality due to too much missing data, so our results cannot determine which type of coffee consumption has an effect on mortality. Finally, the impact of coffee intake on all-cause and CVD mortality in population with sedentary behavior were observed only by a cross-sectional design, which is difficult to determine the causal relationship.

## Conclusions

We found that sedentary behavior was independently associated with higher all-cause and CVD mortality. In contrast, all-cause mortality was reduced in the highest quintile of coffee intake, and the decreased risk for CVD mortality was significantly associated with any amount of coffee consumption. Notably, the results of a joint analysis of this study identified that that the association of sedentary with increased mortality was only observed among adults with no coffee consumption but not among those who had coffee intake. Given that coffee is a complex compound, further research is needed to explore this miracle compound.

### Electronic supplementary material

Below is the link to the electronic supplementary material.

Supplementary Material 1

Supplementary Material 2

## Data Availability

https://www.cdc.gov/nchs/nhanes/index.htm

## Abbreviations

CVD: Cardiovascular disease
NHANES: National Health and Nutrition Examination Survey
NDI: National Death Index
CI: Confidence intervalHR: Hazard ratio
PA: Physical activity
HEI-2015: Healthy Eating Index 2015
BMI: Body mass index
SD: Standard deviation
NA: Not applicable
CRP: C-reactive protein
GPAQ: Global Physical Activity Questionnaire
STROBE: Strengthening the Reporting of Observational Studies in Epidemiology
WWEIA: What We Eat in America
FNDDS: Food and Nutrient Database for Dietary Studies
ICD-10: International Classification of Diseases and Related Health Problems, Tenth Revision
NCHS: National Center for Health Statistics
USDA: United States Department of Agriculture
