# Supplementary material for: Association of daily sitting time and coffee consumption with the risk of all-cause and cardiovascular disease mortality among US adults
Source: BMC Public Health. 2024 Apr 17;24:1069. doi: 10.1186/s12889-024-18515-9 (PMC11022421; doi:10.1186/s12889-024-18515-9)
Supplement: Supplementary file 2 — Supplementary Material 2 [file 12889_2024_18515_MOESM2_ESM.pdf]

Supplemental Online Content

Supplementary Table 1. Weights used in our analysis

Supplementary Table 2. Participants' Characteristics by Coffee Consumption Among US Adults

Supplementary Table 3. Joint Prevalence of Daily Sitting Time and Coffee Consumption Among US Adults

Supplementary Table 4. Association of Daily Sitting Time and with All-Cause and Cardiovascular Disease (CVD) Mortality Among US Adults Stratified by Coffee Consumption Level

Supplementary Table 5. Association of Daily Sitting Time and Coffee Consumption with All-Cause and Cardiovascular Disease (CVD) Mortality in US Adults by excluding deaths that occurred during the first 2-year follow-up

Supplementary Table 6. Joint Association of Daily Sitting Time and Coffee Consumption with All-Cause and Cardiovascular Disease (CVD) Mortality in US Adults by excluding deaths that occurred during the first 2-year follow-up

Supplementary Table 7. Cox regression analysis of the interaction between sedentary behavior and coffee consumption on the risk of all-cause mortality

Supplementary Table 8. Sub-group analysis of the associations between all-cause mortality and daily sitting time

Supplementary Table 9. Sub-group analysis of the associations between all-cause mortality and coffee consumption

**Supplementary Table 1. Weights used in our analysis**	
	NHANES 2007-2018
Formula	1/6 * Fasting Subsample 2 Year MEC Weight
R code	1/6 * wtsaf2yr.lipid
NHANES, National Health and Nutrition Examination Survey	

Supplementary Table 2. Participants' Characteristics by Coffee Consumption Among US Adults

Variable	total	Coffee consumption		Quantiles of coffee consumers		
		non-consumers	Coffee consumers	Q1 [c]	Q2 [c]	Q3 [c]
Age, y	47.1 (0.3)	41.9 (0.3)	51.5 (0.3)	50.4 (0.6)	50.7 (0.51)	53.1 (0.4)
Sex						
Female	5316 (50.0)	2549 (49.7)	2767 (50.2)	1033 (58.0)	932 (50.1)	802 (44.9)
Male	5323 (50.0)	2546 (50.3)	2777 (49.8)	817 (42.0)	931 (49.9)	1029 (55.1)
Race and ethnicity						
Hispanic	2693 (25.3)	1172 (14.3)	1521 (12.5)	658 (20.4)	537 (13.5)	326 (6.2)
Non-Hispanic White	4786 (45.0)	1955 (61.8)	2831 (75.9)	646 (60.3)	928 (75.4)	1257 (87.2)
Non-Hispanic Black	2023 (19.0)	1366 (16.0)	657 (5.5)	277 (8.6)	241 (6.1)	139 (2.9)
Other [b]	1137 (10.7)	602 (7.9)	535 (6.1)	269 (10.8)	157 (5.0)	109 (3.8)
Educational attainment						
<High school	2378 (22.4)	1080 (15.0)	1298 (14.4)	485 (16.7)	443 (14.7)	370 (12.6)
High school	2410 (22.7)	1203 (23.9)	1207 (21.5)	405 (22.7)	384 (19.5)	418 (22.3)
>High school	5851 (55.0)	2812 (61.1)	3039 (64.1)	960 (60.6)	1036 (65.8)	1043 (65.1)
Marital status						
Married	5571 (52.4)	2347 (49.6)	3224 (61.4)	1037 (58.2)	1100 (63.6)	1087 (61.7)
Never married	1908 (17.9)	1278 (25.6)	630 (12.0)	237 (14.2)	230 (13.4)	163 (9.3)
Divorced	1175 (11.0)	514 (9.3)	661 (11.2)	195 (10.1)	195 (8.3)	271 (14.4)
other	1985 (18.7)	956 (15.6)	1029 (15.4)	381 (17.5)	338 (14.7)	310 (14.6)
HEI2015	50.4 (0.3)	49.4 (0.3)	51.4 (0.3)	51.8 (0.5)	51.9 (0.5)	50.6 (0.5)
Family poverty income ratio						
<1.3	3223 (30.3)	1721 (25.2)	1502 (16.8)	541 (19.8)	486 (15.9)	475 (15.5)

1.3-3.5	4061 (38.2)	1945 (36.7)	2116 (35.6)	753 (39.5)	687 (33.4)	676 (34.8)
≥3.5	3355 (31.5)	1429 (38.2)	1926 (47.6)	556 (40.7)	690 (50.7)	680 (49.8)
BMI, kg/m^2						
<25	3037 (28.6)	1485 (30.0)	1552 (29.5)	558 (32.6)	517 (30.8)	477 (26.2)
25-29.9	3554 (33.4)	1591 (31.2)	1963 (34.8)	676 (35.0)	655 (32.6)	632 (36.6)
≥30	4048 (38.1)	2019 (38.9)	2029 (35.7)	616 (32.5)	691 (36.6)	722 (37.2)
Waist circumference	99.4 (0.3)	99.2 (0.4)	99.6 (0.3)	97.3 (0.5)	99.3 (0.6)	101.4 (0.5)
Abdominal obesity						
Yes	6077 (57.1)	2805 (54.8)	3272 (58.3)	1045 (56.0)	1078 (56.0)	1149 (61.9)
Alcohol use						
Never	1369 (12.9)	821 (14.2)	548 (6.5)	264 (10.7)	184 (65)	100 (3.6)
Former	1623 (15.3)	688 (11.5)	935 (13.0)	295 (12.5)	299 (12.1)	341 (14.1)
Mild	3789 (35.6)	1603 (33.1)	2186 (43.1)	722 (42.3)	750 (46.3)	714 (41.0)
Moderate	1664 (15.6)	771 (16.3)	893 (19.2)	278 (17.5)	304 (18.5)	311 (20.9)
Heavy	2194 (20.6)	1212 (24.9)	982 (18.2)	291 (17.0)	326 (16.7)	365 (20.4)
Smoking status,						
Never	5886 (55.3)	3231 (64.5)	2655 (48.1)	1051 (55.3)	957 (52.8)	647 (39.1)
Former	2646 (24.9)	899 (17.7)	1747 (32.1)	522 (30.6)	575 (30.2)	650 (348)
Now	2107 (19.8)	965 (17.9)	1142 (19.8)	277 (14.1)	331 (17.0)	534 (26.1)
Diabetes						
Yes	1474 (13.9)	627 (9.3)	847 (10.7)	297 (10.8)	273 (10.6)	277 (10.6)
Hypertension						
Yes	5585 (52.5)	2472 (44.0)	3113 (51.2)	1020 (49.7)	1045 (50.6)	1048 (52.7)
Cardiovascular disease						

Yes	1138 (10.7)	398 (6.1)	740 (10.8)	251 (10.2)	246 (11.8)	243 (10.3)
Hypercholesterolemia						
Yes	1304 (12.3)	538 (10.6)	766 (14.2)	249 (12.3)	248 (13.4)	269 (16.1)
Cancer diagnosis						
Yes	980 (9.2)	326 (6.4)	654 (12.0)	203 (11.4)	187 (10.6)	264 (13.6)
PA, min/wk						
None (inactive)	2567 (24.1)	1155 (19.6)	1412 (20.7)	507 (24.4)	445 (18.4)	460 (20.3)
0 to <150 (insufficiently active)	1463 (13.8)	665 (12.8)	798 (13.8)	278 (14.1)	261 (13.1)	259 (14.1)
≥150 (active)	6609 (62.1)	3275 (67.6)	3334 (65.5)	1065 (61.5)	1157 (68.6)	1112 (65.6)
Sedentary time						
<4h	2945 (27.7)	1420 (24.3)	1525 (23.3)	579 (26.6)	495 (22.4)	451 (21.7)
4 to 6h	2578 (24.2)	1225 (23.9)	1353 (23.6)	434 (22.6)	477 (24.8)	442 (23.2)
6 to 8h	1675 (15.7)	800 (17.0)	875 (15.7)	298 (15.8)	281 (14.8)	296 (16.4)
≥8h	3441 (32.3)	1650 (34.8)	1791 (37.4)	539 (34.9)	610 (38.0)	642 (38.6)

Abbreviations: BMI, body mass index (calculated as weight in kilograms divided by height in meters squared); PA, physical activity; h, hours; min/wk, minutes per week; NHANES, the National Health and Nutrition Examination Survey.

The descriptive statistics are expressed as mean ± standard deviation and number (percentage) for continuous and categorical variables.

[b] Including American Indian/Alaska Native/Pacific Islander, Asian, and multiracial.

[c] Coffee consumption(g/day) of coffee drinkers was divided into three groups (Q1, Q2 and Q3) by quartile. Q1, Q2 and Q3 are <326, 326-540, >540, respectively.

Supplementary Table 3. Joint Prevalence of Daily Sitting Time and Coffee Consumption Among US Adults

coffee consumption	Daily Sitting Time				
	＜4h	4 to 6h	6 to 8h	＞8h	Total
Q0[a]	13.3	11.5	7.5	15.5	47.9
Q1[b]	5.4	4.1	2.8	5.1	17.4
Q2[b]	4.7	4.5	2.6	5.7	17.5
Q3[b]	4.2	4.2	2.8	6.0	17.2
Total	27.7	24.2	15.7	32.3	100

[a] Q0 means non-coffee consumers.

[b] Coffee consumption(g/day) of coffee drinkers was divided into three groups (Q1, Q2 and Q3) by quartile. Q1, Q2 and Q3 are <326, 326-540, >540, respectively

Supplementary Table 4. Association of Daily Sitting Time and with All-Cause and Cardiovascular Disease (CVD) Mortality Among US Adults Stratified by Coffee Consumption Level

Mortality Outcome	Hazard Ratio (95% CI) [a]			
	**Q0[b]**	**Q1[c]**	**Q2[c]**	**Q3[c]**
**All-Cause**				
Total Sitting Time, h/d				
<4h	1 [reference]	1 [reference]	1 [reference]	1 [reference]
4 to 6h	1.19 (0.70, 2.00)	0.90 (0.50, 1.65)	2.06 (1.21, 3.50)	0.93(0.58,1.50)
6 to 8h	1.06 (0.73,1.55)	1.22 (0.66, 2.25)	1.39 (0.74, 2.59)	0.85(0.44,1.63)
≥8h	1.44 (1.03,2.02)	1.95 (1.11, 3.41)	2.19 (1.38, 3.45)	1.17(0.73,1.87)
p for trend	.03	.002	.02	.51
**CVD**				
Total Sitting Time, h/d				
<4h	1 [reference]	1 [reference]	1 [reference]	1 [reference]
4 to 6h	2.49 (1.02, 6.08)	1.31 (0.52, 3.32)	1.48 (0.42, 5.22)	0.95 (0.29, 3.08)
6 to 8h	2.03 (0.87, 4.71)	1.08 (0.31, 3.77)	0.89 (0.19, 4.10)	0.46 (0.10, 2.07)
≥8h	2.16 (0.99, 4.70)	3.33 (1.14, 9.75)	2.22 (0.66, 7.44)	1.75 (0.66, 4.62)
p for trend	.13	.02	.17	.16

[a] Adjusted for age (years), sex (male or female), race/ethnicity (non-Hispanic white, non-Hispanic black, Hispanic, and other), education attainment (less than high school, high school graduate, above high school), family poverty ratio (<1.30, 1.30-3.49, or ≥3.5), body mass index (BMI; calculated as weight in kilograms divided by height in meters squared) (<25, 25-29.9, and ≥30),smoking status (never, former, current), alcohol use (never, ever, mild, moderate, heavy), Healthy Eating Index-2015, hypertension (yes or no), hypercholesterolemia (yes or no), history of diabetes (yes or no), history of cardiovascular disease (yes or no), and years after first diagnosis of cancer

[b] Q0 means non-coffee consumers.

[c] Coffee consumption(g/day) of coffee drinkers was divided into three groups (Q1, Q2 and Q3) by quartile. Q1, Q2 and Q3 are <326, 326-540, >540, respectively.

.

Supplementary Table 5. Association of Daily Sitting Time and Coffee Consumption with All-Cause and Cardiovascular Disease (CVD) Mortality in US Adults by excluding deaths that occurred during the first 2-year follow-up

Mortality outcome	Death/No.	Weighted death (%)	Hazard ratio (95%CI)		
			Model 1	Model 2	Model 3
**All causes**					
Daily sitting time					
<4h	103/2849	1183990 (2.8)	1 [Reference]	1 [Reference]	1 [Reference]
4 to 6h	129/2489	1695221 (4.1)	1.24 (0.92, 1.66)	1.19 (0.87, 1.63)	1.16 (0.85, 1.58)
6 to 8h	114/1625	1306086 (4.6)	1.32 (0.95, 1.83)	1.20 (0.84, 1.71)	1.16 (0.81, 1.65)
≥8h	261/3338	3404530 (5.4)	1.89 (1.45, 2.45)	1.79 (1.33, 2.42)	1.63 (1.19, 2.22)
***P* for trend**	NA	NA	<.001	<.001	.001
Coffee consumption(g/d)					
Q0[a]	231/4982	2847355 (3.5)	1 [Reference]	1 [Reference]	1 [Reference]
Q1[b]	131/1781	1397355 (5.5)	0.85 (0.64, 1.13)	0.81 (0.61, 1.07)	0.80 (0.60, 1.06)
Q2[b]	116/1780	1504867 (4.8)	0.78 (0.60, 1.00)	0.76 (0.59, 1.00)	0.78 (0.59, 1.02)
Q3[b]	129/1758	1840249 (5.0)	0.79 (0.59, 1.07)	0.67 (0.50, 0.88)	0.66 (0.50, 0.87)
***P* for trend**	NA	NA	.08	.004	.004
**CVD**					
Daily sitting time					
<4h	31/2849	279028 (0.7)	1 [Reference]	1 [Reference]	1 [Reference]
4 to 6h	49/2489	593399 (1.4)	1.74 (0.95, 3.20)	1.67 (0.91, 3.06)	1.65 (0.89, 3.06)
6 to 8h	33/1625	303149 (1.1)	1.28 (0.68, 2.39)	1.18 (0.65, 2.14)	1.14 (0.63, 2.06)
≥8h	76/3338	922251 (1.5)	2.26 (1.35, 3.80)	1.99 (1.18, 3.35)	1.80 (1.06, 3.03)
***P* for trend**	NA	NA	.005	.02	.06
Coffee consumption(g/d)					
Q0[a]	78/4982	929098 (1.1)	1 [Reference]	1 [Reference]	1 [Reference]
Q1[b]	46/1781	411352 (1.6)	0.72 (0.46, 1.13)	0.75 (0.47, 1.18)	0.74 (0.47, 1.16)
Q2[b]	34/1780	376622 (1.2)	0.55 (0.36, 0.85)	0.59 (0.38, 0.93)	0.61 (0.38, 0.96)
Q3[b]	31/1758	380755 (1.0)	0.48 (0.28, 0.84)	0.45 (0.25, 0.80)	0.45 (0.25, 0.79)
***P* for trend**	NA	NA	.002	.001	.002

Abbreviations: h, hours; g/d, grams per day; NA, not applicable; NHANES, the National Health and Nutrition Examination Survey.

**Model 1:** adjusted age.
**Model 2:** multivariable model additionally adjusted for sex (male/female), race and ethnicity (non-Hispanic Black, Hispanic, non-Hispanic White, other race or ethnicity [including American Indian/Alaska Native/Pacific Islander, Asian, multiracial]), education level (<high school, high school, >high school), BMI (<25, 25-29.9, >30), waist circumference, marital status (married, divorced, unmarried) , smoking status (never, former, current), alcohol use (never, ever, mild, moderate, heavy) and Healthy Eating Index-2015 score, family poverty income ratio (<1.30, 1.30-3.49 or >3.5), fasting blood glucose, hypertension (yes/no), history of diabetes (yes/no), hypercholesterolemia (yes/no), cardiovascular disease (yes/no) and history of cancer diagnosis (yes/no).
[a] Q0 means non-coffee consumers.
[b] Coffee consumption(g/day) of coffee drinkers was divided into three groups (Q1, Q2 and Q3) by quartile. Q1, Q2 and Q3 are <330, 330-540, >540, respectively.

Supplementary Table 6. Joint Association of Daily Sitting Time and Coffee Consumption with All-Cause and Cardiovascular Disease (CVD) Mortality in US Adults by excluding deaths that occurred during the first 2-year follow-up

Mortality outcome	Sedentary time	Death/No.	Weighted death (%)	Hazard ratio (95% CI)		
				Model 1	Model 2	Model 3
**All causes**						
Coffee consumers	Sitting time, <6 h/d	145/2751	1718057 (3.9)	1 [Reference]	1 [Reference]	1 [Reference]
	Sitting time, ≥6 h/d	231/2568	3024415 (6.1)	1.60 (1.21, 2.12)	1.43 (1.08, 1.90)	1.32 (0.98, 1.78)
Non-consumers	Sitting time, <6 h/d	87/2587	1161154 (3,0)	1.40 (0.99, 1.97)	1.38 (0.98, 1.96)	1.35 (0.95, 1.92)
	Sitting time, ≥6 h/d	144/2395	1686201 (4.0)	1.86 (1.34, 2.58)	1.93 (1.40, 2.67)	1.83 (1.32, 2.54)
**CVD**						
Coffee consumers	Sitting time, <6 h/d	45/2751	430306 (1.0)	1 [Reference]	1 [Reference]	1 [Reference]
	Sitting time, ≥6 h/d	66/2568	738423 (1.5)	1.66 (1.10, 2.49)	1.47 (0.97, 2.25)	1.35 (0.86, 2.09)
Non-consumers	Sitting time, <6 h/d	35/2587	442121 (1.1)	2.29 (1.29, 4.07)	2.21 (1.21, 4.04)	2.14 (1.14, 3.99)
	Sitting time, ≥6 h/d	43/2395	486977 (1.2)	2.38 (1.39, 4.06)	2.12 (1.24, 3.63)	1.97 (1.14, 3.41)

Abbreviations: h/d, hours per day; NHANES, the National Health and Nutrition Examination Survey.

Model 1: adjusted age.

Model 2: multivariable model additionally adjusted for sex (male/female), race and ethnicity (non-Hispanic Black, Hispanic, non-Hispanic White, other race or ethnicity [including American Indian/Alaska Native/Pacific Islander, Asian, multiracial]), education level (<high school, high school, >high school), BMI (<25, 25-29.9, >30), waist circumference, marital status (married, divorced, unmarried), smoking status (never, former, current), alcohol use (never, ever, mild, moderate, heavy) and Healthy Eating Index-2015 score, family poverty income ratio (<1.30, 1.30-3.49 or >3.5), fasting blood glucose, hypertension (yes/no), history of diabetes (yes/no), hypercholesterolemia (yes/no), cardiovascular disease (yes/no) and history of cancer diagnosis (yes/no).

Model 3: additionally adjusted for PA.

Supplementary Table 7. Cox regression analysis of the interaction between sedentary behavior and coffee consumption on the risk of all-cause mortality

	coef	Hazard ratio	Standard Error	Z value	*P* value [c]
Sedentary behavior (≥8h/d) [a]	0.392995	1.48	0.18	2.397	.017
Coffee consumption [b]	-0.217410	0.80	0.19	-1.118	.26
Sedentary behavior: Coffee consumption	-0.027345	0.97	0.22	-0.119	.91

[a] Refers to sitting for 8 hours or more per day, with less than 4 hours of sitting per day as reference.

[b] Coffee consumption as a binary variable, with non-coffee consumption as reference.

[c] Interaction statistical analysis adjusted for age, sex, race and ethnicity, educational attainment, household poverty-to-income ratio, BMI, waist circumference, marital status, smoking status, alcohol consumption, HEI-2015 score, hypertension, hypercholesterolemia, history of diabetes, CVD, cancer diagnosis, and PA.

Supplementary Table 8. Sub-group analysis of the associations between all-cause mortality and daily sitting time

Variable	Daily sitting time				p for trend	p for interaction
	< 4h	4 to 6h	6 to 8h	> 8h		
Age						.02
≤45	1 [Reference]	2.18 (0.97, 4.93)	0.31 (0.09, 1.08)	1.41 (0.60, 3.33)	.98	
45-65	1 [Reference]	1.31 (0.83, 2.08)	1.43 (0.74, 2.78)	1.47 (0.89, 2.42)	.17	
>65	1 [Reference]	0.91 (0.67, 1.22)	1.00 (0.72, 1.39)	1.69 (1.25, 2.28)	<.001	
Coffee consumption						.996
Yes	1 [Reference]	1.30 (0.94, 1.81)	1.23 (0.82, 1.83)	1.60 (1.21, 2.12)	.002	
No	1 [Reference]	1.18 (0.70, 1.99)	1.13 (0.75, 1.70)	1.33 (0.93, 1.89)	.11	
Sex						.30
Female	1 [Reference]	1.07 (0.70, 1.63)	1.12 (0.78, 1.60)	1.66 (1.17, 2.34)	.003	
Male	1 [Reference]	1.30 (0.93, 1.83)	1.16 (0.79, 1.71)	1.27 (0.94, 1.72)	.27	
Abdominal obesity b						.62
Yes	1 [Reference]	1.15 (0.81, 1.64)	1.18 (0.79, 1.75)	1.59 (1.21, 2.10)	<.001	
No	1 [Reference]	1.36 (0.92, 2.00)	1.21 (0.87, 1.69)	1.37 (0.94, 1.97)	.22	
BMI, kg/m2						.75
<25	1 [Reference]	1.30 (0.86, 1.96)	1.16 (0.73, 1.82)	1.74 (1.14, 2.64)	.02	
25-29.9	1 [Reference]	1.25 (0.85, 1.86)	1.26 (0.80, 1.96)	1.32 (0.91, 1.93)	.19	
≥30	1 [Reference]	1.16 (0.74, 1.81)	1.00 (0.60, 1.68)	1.47 (1.02, 2.11)	.05	
Educational attainment						.35
<High school	1 [Reference]	1.32 (0.89, 1.95)	1.10 (0.72, 1.69)	1.87 (1.33, 2.64)	<.001	
High school	1 [Reference]	1.25 (0.84, 1.86)	1.26 (0.75, 2.11)	1.25 (0.84, 1.85)	.31	
>High school	1 [Reference]	1.20 (0.81, 1.76)	1.13 (0.71, 1.79)	1.51 (1.06, 2.17)	.03	
Race and ethnicity						.05
Hispanic	1 [Reference]	2.50 (1.54, 4.06)	1.80 (0.97, 3.35)	1.83 (1.13, 2.96)	.01	
Non-Hispanic White	1 [Reference]	1.14 (0.86, 1.53)	1.09 (0.78, 1.53)	1.50 (1.17, 1.93)	.002	

Non-Hispanic Black	1 [Reference]	0.87 (0.52, 1.47)	1.30 (0.84, 2.01)	1.21 (0.79, 1.85)	.18	
Other [c]	1 [Reference]	2.62 (0.84, 8.17)	2.13 (0.49, 9.21)	1.59 (0.54, 4.75)	.55	
Marital status						.33
Married	1 [Reference]	1.34 (0.95, 1.89)	1.46 (1.04, 2.05)	1.62 (1.16, 2.27)	.01	
Never married	1 [Reference]	0.68 (0.26, 1.82)	0.34 (0.12,0.93)	0.85 (0.42, 1.72)	.68	
Divorced	1 [Reference]	1.41 (0.71, 2.79)	1.66 (0.66, 4.16)	1.15 (0.54, 2.45)	.82	
other	1 [Reference]	1.18 (0.77, 1.82)	0.92 (0.53, 1.60)	1.47 (1.01, 2.13)	.08	
Alcohol use						.79
Never	1 [Reference]	0.94 (0.52, 1.69)	0.95 (0.48, 1.88)	1.41 (0.80, 2.49)	.23	
Former	1 [Reference]	1.32 (0.95, 1.84)	1.54 (0.97, 2.47)	1.82 (1.30, 2.54)	<.001	
Mild	1 [Reference]	1.11 (0.64, 1.94)	0.95 (0.61, 1.49)	1.38 (0.93, 2.05)	.11	
Moderate	1 [Reference]	1.34 (0.56, 3.18)	0.46 (0.17, 1.25)	1.54 (0.68, 3.51)	.45	
Heavy	1 [Reference]	1.54 (0.81, 2.92)	1.43 (0.56, 3.66)	1.47 (0.78, 2.77)	.38	
Smoking status						.24
Never	1 [Reference]	1.37 (0.93, 2.01)	1.25 (0.84, 1.84)	1.31 (0.92, 1.86)	.24	
Former	1 [Reference]	0.95 (0.67, 1.33)	1.08 (0.68, 1.73)	1.74 (1.23, 2.47)	<.001	
Now	1 [Reference]	1.56 (0.99, 2.46)	1.22 (0.68, 2.19)	1.42 (0.90, 2.24)	.37	
Family poverty income ratio						.96
<1.3	1 [Reference]	1.23 (0.80, 1.89)	1.22 (0.79, 1.87)	1.60 (1.15, 2.25)	.01	
1.3-3.5	1 [Reference]	1.15 (0.82, 1.62)	1.09 (0.76, 1.57)	1.57 (1.16, 2.12)	.01	
≥3.5	1 [Reference]	1.32 (0.72, 2.41)	1.10 (0.55, 2.24)	1.36 (0.83, 2.23)	.30	
Diabetes						.59
Yes	1 [Reference]	0.99 (0.60, 1.62)	1.08 (0.65, 1.78)	1.32 (0.85, 2.07)	.15	
No	1 [Reference]	1.32 (0.97, 1.79)	1.20 (0.85, 1.70)	1.57 (1.20, 2.05)	.002	
Hypertension						.39
Yes	1 [Reference]	1.17 (0.87, 1.59)	1.06 (0.78, 1.45)	1.54 (1.21, 1.95)	<.001	
No	1 [Reference]	1.34 (0.78, 2.30)	1.50 (0.77, 2.90)	1.25 (0.73, 2.12)	.52	

Hypercholesterolemia						.62
Yes	1 [Reference]	1.47 (0.73, 2.96)	1.05 (0.46, 2.43)	1.43 (0.85, 2.41)	.40	
No	1 [Reference]	1.16 (0.91, 1.49)	1.18 (0.88, 1.58)	1.48 (1.17, 1.85)	.001	
Cardiovascular disease						.65
Yes	1 [Reference]	1.00 (0.66, 1.50)	1.12 (0.74, 1.71)	1.52 (1.00, 2.29)	.02	
No	1 [Reference]	1.28 (0.92, 1.78)	1.11 (0.74, 1.65)	1.44 (1.09, 1.90)	.02	
Cancer diagnosis						.35
Yes	1 [Reference]	0.83 (0.48, 1.43)	0.98 (0.60, 1.60)	1.32 (0.78, 2.25)	.16	
No	1 [Reference]	1.36 (1.00, 1.85)	1.25 (0.92, 1.68)	1.51 (1.14, 2.00)	.01	
PA, min/wk						.20
None (inactive)	1 [Reference]	1.06 (0.66, 1.70)	0.96 (0.64, 1.45)	1.63 (1.17, 2.27)	.002	
0 to <150 (insufficiently active)	1 [Reference]	1.63 (0.79, 3.36)	2.34 (1.15, 4.76)	1.72 (1.04, 2.83)	.03	
≥150 (active)	1 [Reference]	1.23 (0.87, 1.75)	1.10 (0.74, 1.64)	1.21 (0.83, 1.75)	.45	

Abbreviations: BMI, body mass index (calculated as weight in kilograms divided by height in meters squared); PA, physical activity; h, hours; min/wk, minutes per week

HRs (95% CIs) were derived from Cox proportional hazards regression models. Covariates were adjusted for sex (male/female), race and ethnicity (non-Hispanic Black, Hispanic, non-Hispanic White, other race or ethnicity [including American Indian/Alaska Native/Pacific Islander, Asian, multiracial]), education level (<high school, high school, >high school), BMI (<25, 25-29.9, >30), waist circumference, marital status (married, divorced, unmarried), smoking status (never, former, current), alcohol use (never, ever, mild, moderate, heavy) and Healthy Eating Index-2015 score, family poverty income ratio (<1.30, 1.30-3.49 or >3.5), coffee consumption, hypertension (yes/no), history of diabetes (yes/no), hypercholesterolemia (yes/no), cardiovascular disease (yes/no), history of cancer diagnosis (yes/no) and PA

[b] Abdominal obesity, men and women were divided into two groups based on their waist circumferences (men, ≥120 cm; women, ≥88 cm)

[c] Including American Indian/Alaska Native/Pacific Islander, Asian, and multiracial

Supplementary Table 9. Sub-group analysis of the associations between all-cause mortality and coffee consumption

Variable	Coffee consumption (g/d)		p for trend	p for interaction
	Non-consumers	Coffee consumers		
Age				.18
≤45	1 [Reference]	0.78 (0.42, 1.46)	.44	
45-65	1 [Reference]	0.60 (0.41, 0.89)	.01	
>65	1 [Reference]	0.91 (0.75, 1.11)	.37	
Daily sitting time				.996
＜4h	1 [Reference]	0.73 (0.48, 1.10)	.14	
4-6h	1 [Reference]	0.85 (0.54, 1.35)	.50	
6-8h	1 [Reference]	0.78 (0.48, 1.29)	.34	
＞8h	1 [Reference]	0.74 (0.57, 0.96)	.02	
Sex				.25
Female	1 [Reference]	0.85 (0.66, 1.10)	.22	
Male	1 [Reference]	0.74 (0.59, 0.93)	.01	
Abdominal obesity [b]				.75
Yes	1 [Reference]	0.78 (0.63, 0.97)	.03	
No	1 [Reference]	0.82 (0.62, 1.08)	.16	
BMI, kg/m^2				.71
<25	1 [Reference]	1.16 (0.86, 1.56)	.32	
25-29.9	1 [Reference]	1.20 (0.85, 1.70)	.30	
≥30	1 [Reference]	1.08 (0.84, 1.40)	.55	
Educational attainment				.34
<High school	1 [Reference]	0.76 (0.60, 0.97)	.03	
High school	1 [Reference]	0.96 (0.68, 1.36)	.82	
>High school	1 [Reference]	0.66 (0.49, 0.89)	.01	
Race and ethnicity				.02
Hispanic	1 [Reference]	0.93 (0.61, 1.43)	.74	
Non-Hispanic White	1 [Reference]	0.83 (0.67,1.04)	.10	
Non-Hispanic Black	1 [Reference]	0.57 (0.40, 0.81)	.002	
Other [c]	1 [Reference]	0.51 (0.22, 1.17)	.11	
Marital status				.68
Married	1 [Reference]	0.73 (0.58, 0.91)	.01	
Never married	1 [Reference]	0.82 (0.39, 1.70)	.59	
Divorced	1 [Reference]	0.93 (0.54, 1.61)	.81	
other	1 [Reference]	0.87 (0.67, 1.15)	.33	
Alcohol use				.78
Never	1 [Reference]	0.73 (0.50, 1.09)	.12	
Former	1 [Reference]	0.80(0.56, 1.14)	.23	
Mild	1 [Reference]	0.75 (0.54, 1.03)	.07	
Moderate	1 [Reference]	0.86 (0.47, 1.58)	.63	
Heavy	1 [Reference]	0.79 (0.42, 1.48)	.46	
Smoking status				.76
Never	1 [Reference]	0.80 (0.64, 0.99)	.04	

Former	1 [Reference]	0.79 (0.58, 1.06)	.11	
Now	1 [Reference]	0.76 (0.52, 1.11)	.15	
Family poverty income ratio				.63
<1.3	1 [Reference]	1.01 (0.75, 1.35)	.96	
1.3-3.5	1 [Reference]	0.78 (0.58, 1.04)	.10	
≥3.5	1 [Reference]	0.66 (0.43, 1.01)	.06	
Diabetes				.07
Yes	1 [Reference]	0.65 (0.50, 0.85)	.002	
No	1 [Reference]	0.83 (0.68, 1.02)	.07	
Hypertension				.22
Yes	1 [Reference]	0.74 (0.62, 0.89)	.001	
No	1 [Reference]	0.92 (0.62, 1.38)	.70	
Hypercholesterolemia				.19
Yes	1 [Reference]	0.62 (0.37, 1.04)	.07	
No	1 [Reference]	0.81 (0.68, 0.96)	.01	
CVD				.64
Yes	1 [Reference]	0.85 (0.64, 1.11)	.23	
No	1 [Reference]	0.76 (0.61, 0.94)	.01	
Cancer diagnosis				.64
Yes	1 [Reference]	0.85 (0.61, 1.19)	.35	
No	1 [Reference]	0.74 (0.61, 0.92)	.01	
PA, min/wk				.89
None (inactive)	1 [Reference]	0.73 (0.58, 0.93)	.01	
0 to <150(insufficiently active)	1 [Reference]	0.86 (0.53, 1.39)	.53	
≥150 (active)	1 [Reference]	0.82 (0.60, 1.12)	.21	

Abbreviations: BMI, body mass index (calculated as weight in kilograms divided by height in meters squared); PA, physical activity; h, hours; min/wk, minutes per week

HRs (95% CIs) were derived from Cox proportional hazards regression models. Covariates were adjusted for age, sex (male/female), education level (<high school, high school, >high school), BMI (<25, 25-29.9, >30), waist circumference, marital status (married, divorced, unmarried), smoking status (never, former, current), alcohol use (never, ever, mild, moderate, heavy) and Healthy Eating Index-2015 score, family poverty income ratio (<1.30, 1.30-3.49 or >3.5), coffee consumption, hypertension (yes/no), history of diabetes (yes/no), hypercholesterolemia (yes/no), cardiovascular disease (yes/no), history of cancer diagnosis (yes/no) and PA

[b] Abdominal obesity, men and women were divided into two groups based on their waist circumferences (men, ≥120 cm; women, ≥88 cm)

[c] Including American Indian/Alaska Native/Pacific Islander, Asian, and multiracial
